# A Review of Eco-Product Value Realization and Ecological Civilization and Its Enlightenment to Karst Protected Areas

**DOI:** 10.3390/ijerph19105892

**Published:** 2022-05-12

**Authors:** Zhenzhen Zhang, Kangning Xiong, Huanhuan Chang, Wenxiu Zhang, Denghong Huang

**Affiliations:** 1 School of Karst Science, Guizhou Normal University, Guiyang 550001, China; zzz3521@163.com (Z.Z.); bjhftw@163.com (H.C.); 201708031@gznu.edu.cn (W.Z.); hdh0503@163.com (D.H.); 2 State Engineering Technology Institute for Karst Desertification Control, Guiyang 550001, China

**Keywords:** ecological products, value realization, ecological civilization, ecological industry, karst protected areas, systematic review of literature

## Abstract

The current ecosystem services of karst protected areas cannot fully enhance human well-being, and the value of eco-products cannot be effectively realized. Research on eco-products and ecological civilization is conducive to the regional sustainability. The results of a statistical analysis of 258 related articles indicate: (1) the number of published articles has increased rapidly after slow growth, indicating that this research field has become a research hotspot and has broad research prospects; (2) the research content mainly involves five aspects, such as eco-product supply, eco-product value realization, eco-industry, ecological civilization, and monitoring and evaluation; (3) the articles research area is mainly distributed in the karst areas with a fragile ecological environment in China and the eco-product value realization and ecological civilization pilot areas; (4) the research frontiers are revealed from four aspects of eco-product supply ability, eco-product value realization, the driving force of eco-product value realization on the formation of eco-industry, model and effectiveness of ecological civilization; (5) it is necessary to deepen the research on the improvement mechanism of eco-product supply capacity, the classification systems and value accounting standards of eco-products, the formation mechanism of eco-industries under ecological threshold constraints and the driving mechanism of eco-industry to ecological civilization.

## 1. Introduction

The 2030 Agenda for Sustainable Development, adopted at the 70th session of the United Nations General Assembly, marks the beginning of a new era for humanity to move toward a sustainable society [1]. Ecological civilization is a new stage in the development of human society, which is related to the well-being of residents and the future of the country [2,3,4]. The ecosystem and socio-economic system constitute a more grand and complex system, in which there is a close relationship between socio-economic elements and ecological elements [5]; the interaction of them can produce eco-products [6]. The term “ecological products” appeared along with the product concept under ecological awareness, and its concept was first expressed by Ren and Yuan [7] in 1992. Natural elements such as air, water and climate were given the attribute of “product” for the first time in the national main functional area planning of China in 2010 [8]. Like agricultural products, industrial products and service products, eco-products are regarded as necessities for human survival and development alongside [9,10]. As the impact of human activities on the earth’s ecosystem continues to expand, ecological economist, Daily, has suggested that the current economic “scarcity” model has changed from abundant natural capital and resources while scarce man-made capital and labor to the opposite [10]; the shortage of high-quality ecological products have become an important bottleneck restricting the sustainable development of the economy and society [11].

The distribution area of modern carbonate rocks in the world reaches 22 million km^2^, accounting for about 15% of the land area [12], affecting the production and life of 1.8 billion people. One of the important goals of karst protected areas is to protect typical karst ecosystems. Karst areas are one of the major ecological fragile zones in the world, and their ecological environment is a hot spot in international geological research today [13,14]. Karst environment has the basic characteristics of high sensitivity to ecological variability, low environmental carrying capacity and small elasticity of disaster tolerance threshold [15]. Affected by natural conditions and interference from human socio-economic activities, the ecosystem in karst areas is very fragile and has a tendency to gradually deteriorate [16].

**Figure 1:**
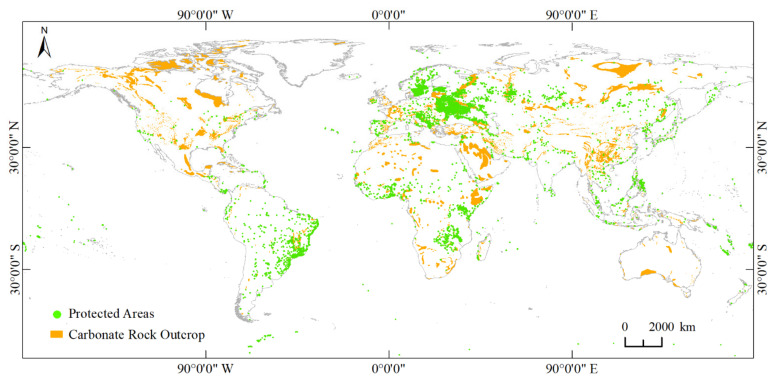
Global distribution of protected areas in karst areas. Protected areas and carbonate rock outcrops are based on data from https://www.protectedplanet.net/en (accessed on 1 April 2022) and https://www.fos.auckland.ac.nz/our_research/karst/index.html (accessed on 1 April 2022).

In March 2022, the Convention on Biological Diversity (CBD) proposed to expand the coverage of protected areas under the global biodiversity framework, and it is planned that the total area of protected areas will reach 30% of the global land area by 2030 [17]. The protected areas in karst areas account for 27.45% of the global protected areas, as of April 2022 (Figure 1). Compared with other regions, ecological civilization construction in karst areas is affected by fragile natural ecosystems and complex social-ecological systems, eco-protection and ecological restoration are more difficult, the residents of communities under the influence of karst culture are less receptive to new things. Ecological civilization construction in karst areas requires more economic, social and cultural capital investment, with characteristics of long cycle, sensitive to environmental changes and easy to rebound. The problem of synergizing the fragile ecological environment with the urgent economic development needs is particularly urgent in karst protected areas [18].

The construction of ecological civilization in karst protected areas is an important measure to protect the eco-environment of karst protected areas and enhance the well-being of karst communities. How to effectively realize the value of eco-products has become a hot issue of common concern for governments and scholars. The purpose of research on eco-product value realization is to solve the externality problem in supply and protect the functionality and integrity of ecosystems [19], and the two are consistent in their objectives. Therefore, the realization of karst eco-protection product value is a practical grasp of ecological civilization construction and plays a vital role in promoting ecological civilization construction in karst protected areas.

Scholars have three main understandings on the connotation of ecological products. The first view equates eco-products with ecosystem services, and believes that they are the well-being that natural ecosystems provide to humans [20,21]. The second view holds that eco-products are produced jointly by man and nature [9]. The third view holds that eco-products also include eco-label products, green label products, eco-designed products, eco-friendly products and pollution-free products [22]. With the deepening of ecological civilization, high-quality eco-products are becoming increasingly scarce, and the shortage of eco-products has become an important bottleneck limiting sustainable economic and social development [11]. In recent years, the concept that has been widely recognized by scholars has been proposed by Zhang et al. [9]. They believe that eco-products are the final products or services provided by ecosystems for human well-being through biological production and interaction with human production. However, the research on eco-product value realization is still in the exploratory stage. Scholars have not yet reached a consensus on the concept and classification of eco-products, the value realization mechanism lacks in-depth discussions, and the driving mechanism of eco-products on ecological civilization is still unclear. Those factors affect the regional ecological and economic sustainability. Therefore, we present findings from our literature review on eco-product value realization and ecological civilization, and propose key scientific issues that need to be solved along with future research directions.

## 2. Data and Methods

**Figure 2:**
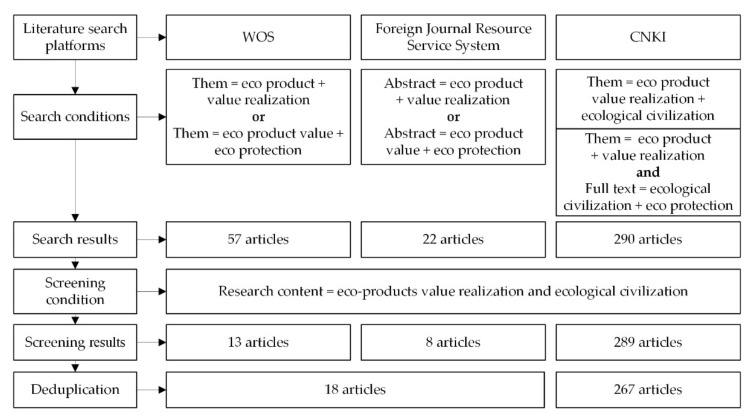
The process of the literature search and screening.

To identify relevant studies, a search was conducted based on the platforms including Web of Science (WOS) (https://www.webofscience.com), Foreign Journal Resource Service System (http://fpd.juhe.com.cn/) and China National Knowledge Infrastructure (CNKI) (https://www.cnki.net/). The searching date was on 12 November 2021. Figure 2 showed the process of literature search and screening.

First, English articles were obtained based on WOS and the foreign journal resource service system. In WOS, with “theme” as the search item, “eco product” and “value realization”, “eco product value” and “eco protection” as the search terms, 57 documents were found. In the foreign journal resource service system, with “abstract” as the search item, “eco product” and “value realization”, “eco product value” and “eco protection” as the search terms, 22 articles were found. Secondly, Chinese articles were obtained based on CNKI. In CNKI, with “them” as the search item, “eco product value realization” and “ecological civilization” as the search terms for the first search, with “them” as the search item, “eco product” and “value realization” as the search terms, and with “full text” as the search item, “ecological civilization” and “eco protection” as the search terms for the second search, 290 articles were found.

Then, according to the research content of eco-product value realization and ecological civilization, irrelevant articles were screened out: 13, 8 and 289 articles were obtained from WOS, the foreign journal resource service system and CNKI, respectively.

After deduplication, we finally obtained 18 English articles and 267 Chinese articles. The top 10 contributors in number of articles of the topic are Xiahui Wang (6), Linbo Zhang (5), Yihong Zhou (5), Weiming Li (5), Shuilin Qiu (4), Hongxing Zhang (4), Zhiyun Ouyang (4), Huiyi Yu (4), Shuzhong Gu (4) and Jinnan Wang (3). When calculating the number of published articles by authors, all authors in the articles were accounted regardless of the author’s order.

## 3. Results

### 3.1. Annual Distribution

**Figure 3:**
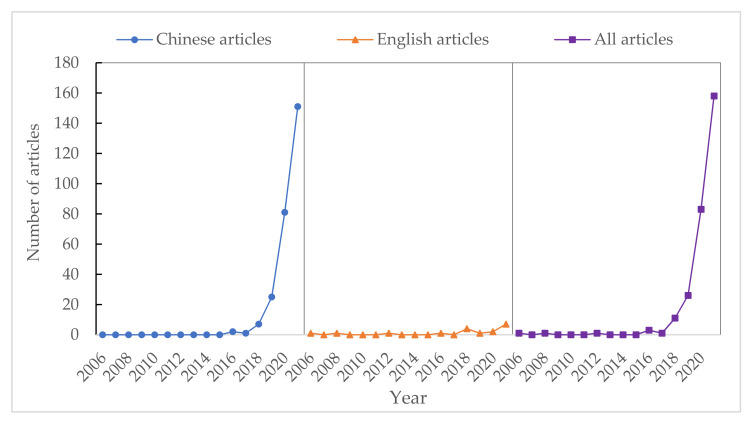
Annual distribution of articles.

In 1990, ecological civilization was first systematically proposed in Ecological Awareness and Ecological Civilization [23]. In 2005, eco-product value realization was first researched in The Price Composition of Ecological Public Products and Its Realization Mechanism [24]. In 2005, the thesis that “lucid waters and lush mountains are invaluable assets” was put forward by Jinping Xi for the first time, which explained the unity of opposites between economic development and environmental protection, and opened the prelude to the research on eco-product value realization and ecological civilization. The research on eco-product value realization and ecological civilization began to sprout in 2006 and has increased rapidly since 2018 (Figure 3). In general, the number and trend of publications in Chinese articles are the same as those in all articles. The research can be divided into two stages. At the first stage (2006–2017), the annual number of articles were not more than 10, as it was the sprouting stage. The second stage (2018–2021) showed the trend of rapid growth, indicating a broad research prospect in this research field.

### 3.2. Content Distribution

**Figure 4:**
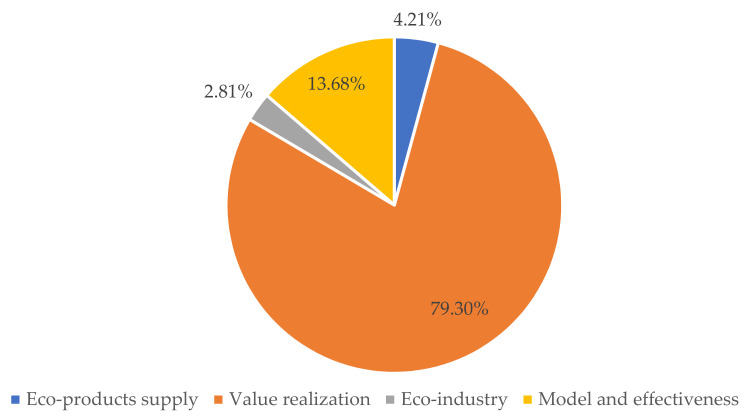
Articles by content.

The contents of the articles are shown in Figure 4. All the identified articles are classified and summarized in terms of eco-product supply, value realization, eco-industry, ecological civilization and evaluation. Among the articles, there are only 12 relevant articles on eco-product supply, mainly qualitative research from the perspective of ecological space planning and control, eco-product supply and demand. The mainstream of research in this field, accounting for 79.30% of the total number of articles, mainly focuses on value accounting, the value realization model, path, mechanism and ecological compensation. There are eight relevant articles on eco-industry, mainly concerning theoretical framework, development path and strategy and industrial development evaluation. Studies on ecological civilization account for 11.58% of the total articles, mainly involving the ecological civilization model and path, relationship between the theory of “lucid waters and lush mountains are invaluable assets” and eco-product value realization, ecological civilization effectiveness and strategies. Studies on monitoring and evaluation are the fewest, with only six, mainly using ecological efficiency, EcoDP and other methods to evaluate the level of regional green development. In general, due to the short period of time for studies on eco-product value realization and ecological civilization, most of the studies are still at the stage of summarizing practical experience and researching theoretical models, paths, frameworks and strategies. A small amount of relevant quantitative research focusses on value accounting and monitoring and evaluation.

### 3.3. Institutions Distribution

We conducted an interpretation and analysis of the distribution of organizations by using the occurrence frequency of each institution of related articles as the basis, and then sorted them from high to low. The statistical results show that the studies are mainly distributed in the following three types of research institutions. (1) Institutions that have long been engaged in the related research on ecological civilization and sustainable economic development, and have good relevant research foundation and experience, such as: the Chinese Academy of Natural Resources Economics, Environmental Planning Institute of the Ministry of Ecology and Environment and Development Research Center of the State Council. (2) Institutions in finance and resources directly related to the subject of the research, such as Lanzhou University of Finance and Economics, Central University of Finance and Economics and Hebei University of Economics and Business. (3) Institutions in the areas where the national eco-product value realization mechanism pilots are located, such as: Shandong University, Zhejiang University and Lishui College. Moreover, senior colleges and universities with majors related to agriculture and forestry are also the main camp of research institution articles with related studies, such as China Agricultural University and Beijing Forestry University, Renmin University of China. In general, research foundation and experience, professional connection and geographical advantages are the main factors affecting the distribution of articles research institutions.

### 3.4. Research Stages

Table 1 shows the annual distribution of articles. The related studies started in 2006, and thus far, have a history of nearly 15 years. According to the research background of the changes in eco-product supply, value realization, eco-industry, ecological civilization, monitoring and evaluation during this period of time, studies about eco-product value realization and ecological civilization are divided into two stages, namely the sprouting stage and rapid growth stage.

## 4. Main Developments and Landmark Achievements

### 4.1. Eco-Product Supply

The “meta-rule” of territorial spatial planning is the preservation and appreciation of natural resources [25]. At the planning level, functional divisions can be optimized based on the marketization degree and product production category. The content of use control can be enriched with “eco-product production license lines”. The market-oriented allocation of elements can be improved with the guidance of system coupling [26]. Wang et al. [27] believe that in the main agricultural product producing areas and key ecological function areas, it is necessary to increase the power of financial transfer payments, and establish and improve the horizontal ecological compensation mechanism in the river basin to enhance the supply capacity of eco-products. Zhao et al. [28] found the mutually beneficial relationship between greenway tourism and agricultural heritage protection through participatory observation, in-depth interviews and questionnaire surveys. The implementation of greenways has driven the rise and rapid development of tourism, increased the income of villagers in agricultural heritage sites, and stimulated their enthusiasm to protect the environment and sell agricultural products. Karst protected areas should pay attention to the control and design of ecological space, maintain the service function of the ecosystem, and ensure the supply capacity from the source.

Ecological design and product full-cycle environmental management have become important links in the research and development of eco-products. To quantitatively evaluate eco-product design, Ng [29] proposed a simplified life cycle assessment and ant colony optimization algorithm to quantitatively assess product-oriented environmental impact and identify the product assembly sequence with the lowest environmental impact. The product-based environmental management system addresses the impact of hardware products on the environment, especially the impact of wireless hardware products on the environment during the entire product life cycle [30]. In the research and development of karst eco-protection products, the environmental impact of the entire life cycle of eco-products should be fully considered, and eco-product design and product-based environmental management systems suitable for karst eco-protection areas should be explored. Moreover, a third-party quantitative evaluation system should be established.

The environmental cost in the process of economic development has caused a prominent contradiction between the supply and demand of eco-products. It is urgent for us to improve the ecological environment, increase the total supply of eco-products and enhance the supply capacity [31]. The supply capability depends not only on the region’s own endowments of ecological factors, but also on the influence of social subjects such as governments, markets and enterprises [32]. Therefore, to enhance the endogenous power of eco-product supply, it is necessary not only to open up the channels for realizing economic, social, cultural and ecological value, to improve the complete benefits of supply actions, but also to fully consider the intergenerational impact of eco-products and balance the current benefits and intergenerational benefits of eco-product supply [33]. For eco-products with the characteristics of private goods, market-oriented supply is an effective supplement to government supply [34]. Karst protected areas can optimize the supply structure from regional eco-resource endowment protection, endogenous power improvement and supply system improvement.

The core of expanding consumption demand of eco-products is to coordinately promote the whole of society to form ecological values, green lifestyles and green consumption models [5]. The use of eco-labels [35] and eco-certification [36] can reduce the environmental impact at the source, and stimulate the pride of consumer groups with environmental values when purchasing raw eco-products. Using marketing and branding to promote and sell eco-products is conducive to the development of the eco-product market. The market-oriented supply methods of eco-products mainly include economic transactions in the direct market, industrialized operation of ecological capital and ecological purchases [37]. Karst protected areas can use ecological labels, ecological certification, economic transactions (such as emissions trading, water trading, carbon emissions trading), ecological capital industrialization, ecological purchasing and other means to develop eco-products markets and optimize the market-oriented supply model of eco-products.

### 4.2. Value Realization

The calculation method of eco-product value usually adopts the calculation method of ecosystem service value, which is divided into two stages of functional volume accounting (also called physical volume accounting) and monetary value volume accounting according to the studies by Daily et al. and Ouyang et al. [10,38,39]. Usually, the process and methods of eco-product value accounting are as follows: (1) determine the spatial scope and accounting year of eco-product value accounting; (2) clarify the types and distribution of ecosystems; (3) compile the list of eco-products in the ecosystem; (4) collect data and supplementary surveys; (5) carry out physical accounting of eco-products; (6) carry out value volume accounting of eco-products; (7) calculate the total value of regional eco-products.

Ecological banks such as forest banks, wetland banks and water banks are new platforms for value realization. These ecological banks can promote the capitalization and make eco-products become productive forces. So, they can change resources into assets. The operation process of ecological banks includes resource investigation, project planning, value evaluation, circulation reserve and operation [40]. These ecological banks focus on providing raw material base, heavy assets, ecology and market services. Through these banks, the ecological resources can be transformed into assets.

Eco-products have diverse and complex characteristics, so the value realization paths are also different. The property rights of purely public eco-products (such as clean water) are common. The economic value of them, which is difficult to realize through market transactions, mainly relies on governmental paths. Value payments of them are in the form of transfer payments, ecological compensation [41] and special government funds to support eco-protection. Quasi-public eco-products (such as public forest land) can realize value under government control through taxes or eco-resource equity trading [42]. Operational eco-products (such as ecotourism) can realize their value directly through market transactions. The payment form is the price of the products themselves. The ecological premium of ecological material products generally requires credible third-party certification and evaluation.

### 4.3. Eco-Industry

Entering the era of ecological civilization, the classical division of labor among the three industries can no longer meet the needs of industrial development in the era of ecological civilization. Ye and Han [43] proposed to define waste reuse as the quaternary industry to show the importance of environmental production. However, it is too narrow to define eco-environmental production as waste reuse only. Wang et al. [10] constructed the quaternary industry of eco-products from the perspective of eco-products, proposing that eco-products are the “fourth category” of products. They further analyzed the formation mechanism and components of the quaternary industry, and constructed an evaluation indicator system. The theory of the quaternary industry innovatively elevates the industries formed by eco-products to a new height alongside traditional industries. It is of milestone significance in promoting the eco-product value realization and ecological civilization. However, the theory is still at a preliminary stage, and the boundary of the quaternary industry is still unclear, leading to confusion between eco-products and traditional products. The inputs, intermediate goods and final products should be clarified. A more systematic accounting system that avoids double counting should be built [10].

Eco-resources must be fully relied on to realize ecological industrialization, and regional characteristics must be found to promote industrial ecologicalization [44]. Both ecological industrialization and industrial ecologization are based on eco-products and eco-industries. Industrial ecologization is the process of realizing the resource-saving, environment-friendly and eco-protection of industry. Ecological industrialization is the process of large-scale production and value realization of eco-products. Therefore, ecological industrialization is mainly applicable to nature reserves with good eco-environments [45]. The production of eco-products promotes ecological industrialization, which is one of the endogenous driving forces for value realization. Industrial eco-transformation promotes the improvement of ecological concepts and the increase in demand for eco-products, which is the external driving force for eco-product value realization. Karst protected areas should give full play to the advantages of eco-resources and combine eco-products with regional natural resources and ethnic cultural resources. Then ecological industrialization can be conducted in core areas and industrial ecologicalization can be conducted in buffer zones.

The eco-industry chain of protected areas is the input–output relationship of material, value and information. Based on the input–output perspective of eco-products, the eco-industry chain structure model includes four links: the eco-product industry chain, the derivative industry chain, the product demand market and the supporting industry chain [46]. The health of eco-industry chain is usually expressed by the flexibility of eco-industry chain. The flexibility of the eco-industry chain is the potential for the long-term operation of the eco-industry chain, especially the resilience against external changes [47], similar to the concept of ecosystem resilience. The core area of the nature reserve is the main production area of eco-products, and the buffer zone and experimental area are the main production areas of derivatives. The sharing of eco-products and the development of derivatives are the basis for the construction of the eco-industry chain. Therefore, the trade-off and synergy between eco-products and derivatives is the dominant factor that determines the flexibility of the eco-industry chain. Karst protected areas have to weigh the balanced relationship between eco-products and derivatives, the eco-product industry chain and the derivative industry chain, in the value realization and eco-industry development.

### 4.4. Model and Effectiveness

Due to the differences in natural and socio-economic resource endowments, the specific models and paths for ecological civilization vary from place to place. Bao [48] believes that building a consumption pattern that is conducive to saving energy resources and protecting the ecological environment is an important path for ecological civilization. Liao et al. [49] summarized the ecological civilization model of Fujian Province in China into a collaborative innovation model and a green development-oriented model. Jin [50] divided China’s ecological civilization practices into four types: dual-value-limited, ecological-value-oriented, Pareto-optimal and economic-value-oriented from the perspective of economic value and ecological value. Karst protected areas must adhere to the orientation of ecological value in the construction of ecological civilization and maintain and enhance the ability to create ecological value in eco-protection and eco-restoration.

The development level of ecological civilization is usually measured by eco-efficiency [51], ecological domestic product (EcoDP) [52] and the dual-benchmark incremental method [53]. Eco-efficiency is a direct synthesis of two universally recognized indicators, GDP and ecological footprint. It is simple in principle, convenient in calculation and easy to apply. EcoDP incorporates the ecological content into the evaluation framework of regional sustainable development level and improves the evaluation system of sustainable development. In the dual-benchmark incremental method, each evaluation value in this method has its practical significance, not only can the scores of different cities be compared, but also the scores of each indicator in this indicator system can be compared with each other, and the urban ecological civilization level can be divided by the score interval, providing knowledge for managers to make decisions. When evaluating the effectiveness of ecological civilization, karst protected areas can build karst characteristic evaluation models and methods based on the vulnerability of karst social-ecosystems, and incorporate karst eco-protection and eco-restoration into the evaluation system.

## 5. Key Scientific Issues to Be Solved

### 5.1. The Mechanism for Improving the Supply Capacity of Eco-Products

The contradiction between the supply shortage of eco-products and the demand for high-quality eco-products has become increasingly prominent [54]. It is urgent to improve the supply capacity of eco-products and create a good atmosphere for all parties to participate in ecological environmental protection and restoration [55]. Current studies on supply capacity have mostly stayed at the level of upgrading paths and strategies, such as eco-spatial planning and design, eco-labeling, eco-certification and trading mechanisms. Some scholars have also discussed the endogenous dynamic mechanism for eco-product supply from the perspective of market players, but none of them has gone deeper into the landscape pattern and ecosystem service level.

From the perspective of eco-product value formation, the biological and physical structure of the ecosystem and the process of material, energy and information flow form the ecosystem functional units. Each functional unit ensures the supply of ecological material products and ecological service products. Under certain conditions, when material products and service products of the ecosystem meet human needs, the monetization value assessment can be conducted. Coupled with the design of a reasonable transaction mechanism, the value of eco-products can be realized [21]. Therefore, the improvement of the eco-product supply capacity should comprehensively consider natural ecosystems and social ecosystems, and analyze the mechanism for improving the supply capacity of eco-products based on “landscape pattern-structure and process-ecosystem function-consumer demand” from two perspectives: eco-products and ecological market.

Karst protected areas should analyze the particularity of consumers’ demand for eco-products under the influence of karst culture on the premise of understanding the impact of karst fragmented landscape pattern and dual structure on ecosystem functions, and then clarify the mechanism for improving the supply capacity of karst eco-protection products.

### 5.2. The Classification Systems and Value Accounting Standards of Eco-Products

Scholars have different views on the concept and classification of eco-products. Due to the differences in the concept of eco-products, the classification systems and value accounting standards of eco-products are also different, which directly affects the accuracy and comparability of the value accounting, and hinders the process of value realization. Many scholars directly use Gross Ecosystem Product (GEP) to calculate the value of eco-products, and include agricultural products, forestry products, animal husbandry products and fishery products into eco-products [5,56]. GEP is a good method to calculate the value of ecosystem service, but not perfect for the value of eco-products. The indicator in this method overlaps eco-products and traditional products and may overestimate the eco-products value. It is of great importance to clarify the concept of eco-products, clarify the boundary between them and ecosystem services, eco-friendly products, eco-label products, eco-design products and traditional agricultural, industrial and service industry products, and build the classification system and value accounting standards.

### 5.3. The Formation Mechanism of Eco-Industries under the Constraints of Eco-Thresholds

With rapid economic and social development, many protected areas are facing the dual pressure of eco-protection and economic development. Eco-industries give full play to the advantages of natural resources and economic resources have strong survival, expansion and competitiveness. It is necessary to establish a coupling model between ecological carrying capacity and eco-industries, and clarify the interaction between the two. The ecological control mechanism for the formation and optimization of eco-industries is also important. The first is to analyze the unique attributes of karst protected areas, improve the accounting indicators and methods of GEP and calculate the functional and monetary value of karst eco-products. The second is to collect and optimize the fragmented management rights and use rights of eco-resources, expand the paths for value realization and explore the value-added mechanism of eco-products. The third is to build trading systems including eco-products certification system, clarify the driving factors for the formation of eco-industries, optimize the industrial structure and clarify the formation mechanism of the eco-industries.

### 5.4. The Driving Mechanism for Eco-Industries on Ecological Civilization

In the critical period when human society is entering the ecological epoch and realizing the improvement of ecological environment quality from quantitative change to qualitative change, the important position of eco-industries in strongly promoting ecological civilization is becoming increasingly more prominent [57]. Due to historical reasons, special natural environments and socio-economic characteristics, as well as the special status of conservation priority, there are problems such as ambiguous driving force and narrow path for eco-industries to promote ecological civilization in karst reserves. Around the driving mechanism of eco-industries in karst protected areas for ecological civilization, research can be conducted in terms of eco-culture, eco-economy, eco-responsibility, eco-system and eco-security.

For example, based on ecological industrialization of karst reserves and industry ecologicalization of buffer zones, the driving mechanism for ecological industries extension on the eco-economic system is to be explored. Based on eco-cultural gene mapping, eco-brand culture shaping and ecological value enhancement, the driving mechanism for ecological culture inheritance on the eco-cultural system is to be elucidated. The driving mechanism for the multi-subject participation of eco-industry management on the eco-responsibility system is to be revealed. The driving mechanism for trade-offs, synergies and compensation of eco-industrial benefit distribution to the eco-system is to be clarified. The inner logic for the five systems of ecological civilization is to be analyzed, the driving framework for eco-industries to the eco-security system can be conducted and the driving mechanism for eco-pattern formation to the eco-security system can be proposed.

## 6. Conclusions and Future Research

In this paper, we conducted a systematic literature review by analyzing 258 articles retrieved from the Web of Science, CNKI and Foreign Journal Resource Service System of Guizhou Normal University Library. The main conclusions are as follows: (1) studies on eco-protection product value realization and ecological civilization are increasing rapidly, showing a broad research prospect; (2) among the studies on eco-product supply, value realization, eco-industry, ecological civilization and monitoring and evaluation, the studies on eco-product value realization are most common, mainly focusing on mechanisms and paths for value realization; (3) the related research has mainly been conducted in China. After analyzing the main developments and landmark achievements, the paper proposed four scientific issues to be addressed.

The reasons for more literature written in the Chinese language than in English may be as follows: (1) the concept of ecological products has been put forward in China by the Chinese, and some pilot projects in China have formed models that are worth learning from; (2) international scholars use the concept of ecosystem services more than ecological products. The eco-products related academic research is still in the exploratory stage. Some scholars even confuse the concepts of eco-products and ecosystem service, believing that the two are the same.

In fact, the two concepts are different. Ecosystem service has been widely studied internationally. The concept of eco-products was proposed based on ecosystem service, but is an extension to it. Ecosystem services emphasize the benefits of natural ecosystems to humans, while eco-products emphasize the human–nature life community. Ecosystem service value emphasizes the value of ecology and arouses the awareness of human beings to protect nature and protect ecology. The value of eco-products emphasizes the value of products and services jointly created by man and nature, and emphasizes the value created by the behavior of human beings to protect and restore ecology.

The future directions of eco-products and ecological civilization can be conducted based on the following aspects: (1) the mechanism of improving the supply capacity of eco-products based on eco-products (ecosystem structure and process) and the ecological market (consumer demand); (2) the classification systems and value accounting standards of eco-products; (3) the formation mechanism for ecological industries under the constraints of ecological thresholds based on the carrying capacity of ecosystems; (4) the driving mechanism for eco-industries on ecological civilization. These future research topics are especially suitable for protected areas, such as natural world heritage, geoparks, nature reserves and scenic spots, due to the high-grade eco-products in these areas.

**Figure 5:**
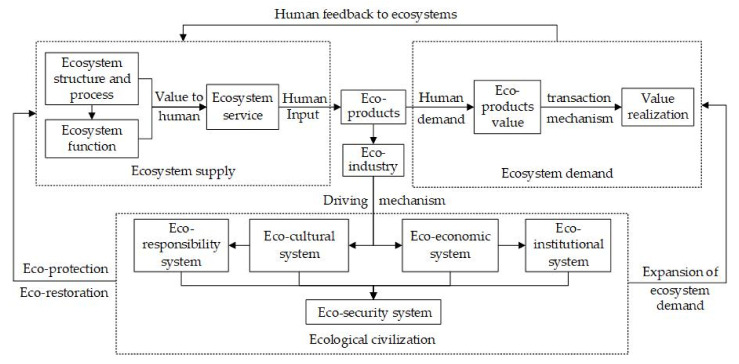
Research ideas on the eco-product value realization and ecological civilization.

Figure 5 shows the research ideas based on the three perspectives of ecosystem supply, ecosystem demand and ecological civilization and their synergistic relationship. The structure and process of the ecosystem determine the function of the ecosystem, and its value to humans is the ecosystem service, which constitutes the supply of the ecosystem. On the basis of the ecosystem service, human input is added to produce eco-products. When eco-products meet human needs, they have values. The value of ecological products can be realized through transaction mechanisms. Those above constitute ecosystem demand. Ecosystem supply acts on ecosystem demand through eco-products, and ecosystem demand acts on ecosystem supply through human feedback on the ecosystem.

Through the study of the characteristics and supply of the ecosystem, the attributes of eco-products, the mechanism for improving supply capacity and the mechanism for value realization are clarified. The industrialization of eco-products produces eco-industries, which directly drives the construction of the eco-economic system and eco-cultural system, and then promotes the construction of the eco-institutional system and eco-responsibility system, thereby building the eco-security system and realizing the goal of ecological civilization.

Ecological civilization acts on ecosystem supply through ecological protection and ecological restoration, and acts on ecosystem demand through ecological demand expansion. Through research on the formation mechanism for the eco-industry and the driving mechanism for eco-industry to ecological civilization, the model and paths of ecological civilization based on ecological protection can be conducted to provide the basis for the construction of ecological civilization and sustainability in karst protected areas.

## Figures and Tables

**Table 1:** Division of the research stages.

Research Stage	Main Characteristics	Background
Sprouting stage (2006–2017)	There are less than five papers per year. A few related articles were consulted and there were none in some years; search contents mostly concentrated on the design of eco-products and ecological civilization frame, and descriptive theoretical research and practical experience summaries were in the majority.	In 2005, Jinping Xi first proposed that “lucid waters and lush mountains are invaluable assets”, which opened the prelude to the research on the eco-product value realization and ecological civilization. Some scholars began to study the related concepts.
Rapid growth stage (2018–2021)	The number of articles has increased rapidly, with more than 10 papers per year. Eco-product value realization mechanism is the focus of the research at this stage. Quantitative research and exploration of ecosystem gross product (GEP) and eco-product value accounting began to appear.	China has carried out pilots and experimental areas for eco-product value realization mechanism and ecological civilization in many cities, and issued articles to promote their further implementation. High-quality eco-products have become scarce resources, and the coordinated development of ecology and economy has attracted increasingly more attention.

## Data Availability

The data presented in this study are openly available in [China National Knowledge Infrastructure (CNKI)] at [https://www.cnki.net/], [Foreign Journal Resource Service System of Guizhou Normal University Library] at [http://fpd.juhe.com.cn/] and [Web of Science (WOS)] at [https://www.webofscience.com].
